# Association of sudden sensorineural hearing loss with dementia: a nationwide cohort study

**DOI:** 10.1186/s12883-021-02106-x

**Published:** 2021-02-25

**Authors:** Shu-Yu Tai, Cheng-Ting Shen, Ling-Feng Wang, Chen-Yu Chien

**Affiliations:** 1 Department of Family Medicine, School of Medicine, College of Medicine, Kaohsiung Medical University, Kaohsiung City, 807 Taiwan; 2 Department of Family Medicine, Kaohsiung Medical University Hospital, Kaohsiung Medical University, Kaohsiung City, 807 Taiwan; 3 Department of Family Medicine, Kaohsiung Municipal Ta-Tung Hospital, Kaohsiung Medical University Hospital, Kaohsiung Medical University, Kaohsiung City, 801 Taiwan; 4 Department of Otorhinolaryngology, School of Medicine, College of Medicine, Kaohsiung Medical University, Kaohsiung City, 807 Taiwan; 5 Department of Otorhinolaryngology, Kaohsiung Medical University Hospital, Kaohsiung Medical University, Kaohsiung City, 807 Taiwan; 6 Department of Otorhinolaryngology, Kaohsiung Medical University Hospital, No. 100, Tzyou 1st Rd., Sanmin District, Kaohsiung City, 80708 Taiwan (Republic of China)

**Keywords:** Sudden sensorineural hearing loss, Dementia, Nationwide cohort study, Female, Older adults

## Abstract

**Background:**

Impaired cochlear blood perfusion and microvascular damage can cause sudden sensorineural hearing loss (SSHL), which is a potential risk factor for dementia. This study explored the association between SSHL and dementia.

**Methods:**

This retrospective cohort study used a random sample of 1000,000 individuals from Taiwan’s National Health Insurance Research Database. We identified 3725 patients newly diagnosed with SSHL between January 1, 2000, and December 31, 2009, and propensity score matching according to age, sex, index year, comorbidities, and medications was used to select the comparison group of 11,175 patients without SSHL. Participants were stratified by age (<65 and ≧65 years) and sex for the subgroup analyses. The outcome of interest was all cause dementia (ICD-9-CM codes 290.0, 290.4, 294.1, 331.0). Both groups were followed up until December 31, 2010, for diagnoses of dementia. Cox regression models were used to estimate the hazard ratio (HR) of dementia.

**Results:**

During the average 5-year follow-up period, the incidence rate of dementia in the SSHL cohort was 6.5 per 1000 person-years compared with 5.09 per 10,000 person-years in the comparison group. After adjustment for potential confounders, patients with SSHL were 1.39 times more likely to develop dementia than those without SSHL (95% confidence interval = 1.13–1.71). When stratified by patients’ age and sex, the incidence of dementia was 1.34- and 1.64-fold higher in patients with SSHL aged ≥65 years (*P* = .013) and in women (*P* = .001), respectively, compared with the comparison group. Women with SSHL who were < 65 years old had the highest risk (2.14, 95% CI = 1.17–4.11, *P* = .022). In addition, a log-rank test revealed that patients with SSHL had significantly higher cumulative incidence of dementia than those without SSHL (*P* = .002).

**Conclusions:**

Patients with SSHL, especially women aged < 65 years, were associated with higher risk of dementia than those without SSHL. Thus, clinicians managing patients with SSHL should be aware of the increased risk of dementia.

## Background

The proportion of older adults (≧65 years) in Taiwan has been gradually increasing—from 4.1% in 1980 to 14.05% in 2018 and is estimated to reach 20% in 2025 [1], which will make Taiwan a super-aged society [2]. The global population, in general, is rapidly aging, and the global prevalence of dementia will therefore have significantly increased by 2050, with an estimated prevalence of 59% in Asia [3], indicating a potential dementia epidemic. However, no measures exist for preventing cognitive decline in older adults [4, 5]. Therefore, it is crucial to determine which patients are at risk of dementia.

Any type of hearing loss (HL), especially when related to aging, is a risk factor for cognitive decline, cognitive impairment, and dementia [6]. Although the causal relation between HL and cognitive decline remains unclear [7], the two conditions may have a common etiology, as both are related to vascular dysfunction and extended physiological decline. Aged-related HL (ARHL) is also linked to multiple indicators of functional decline [8]. Another theory postulates that HL in older adults can lead to a lower quality of life, social isolation, depression, and disability, all of which increase the risk of dementia [9].

Sudden sensorineural HL (SSHL) is defined as a sensorineural HL of ≥30 dB in three sequential frequencies within 3 days [10]. It is an urgent neurotology disorder requiring immediate identification and treatment [11]. SSHL occurs at any age but usually affects people in their 40s and 50s [12], with an annual incidence of 5–20 per 100,000 people per year in developed countries and with an equal sex distribution [13]. It can occur in isolation or as part of a systemic disorder. Four potential causes of SSHL include vascular disorder, autoimmune diseases, inner ear membrane rupture, and viral or bacterial infections. Currently, the vascular basis is the favored explanation; the abrupt onset of SSHL, such as by cerebral stroke or acute myocardial infarction [14, 15], may be correlated with a vascular event also contributing to cognitive impairment. For example, Phillips et al. [16] described a case of SSHL coupled with cerebral autosomal dominant arteriopathy, subcortical infarcts, and leukoencephalopathy, including intermittent cerebrovascular events, migraines, and dementia.

Here, we used Taiwan’s national population-based database to examine whether SSHL contributes to the development of dementia. Our results have practical implications for clinicians and may guide the development of preventive measures for dementia in patients with SSHL.

## Methods

### Data source

In this cohort study, data were collected from the National Health Insurance (NHI) Research Database (NHIRD) [17], which includes data on inpatient, outpatient, ambulatory care, and previous medical conditions dating from January 1, 1997, to December 31, 2010. Taiwan’s NHI program, established in 1995, is a government-operated single-payer insurance system that covers the health care of the entire nation in hopes of eradicating social problems caused by poverty and disease. As of 2019, 99.9% of Taiwan’s population is participating in the NHI program.

### Study design and population

**Fig. 1:**
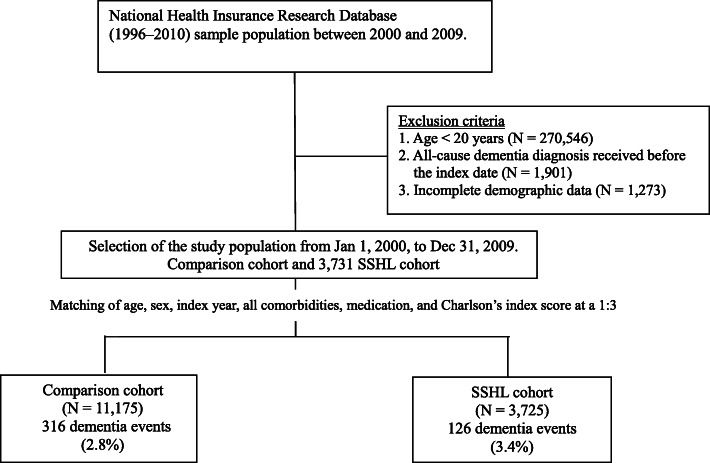
Flowchart of the study design

A population-based retrospective matched cohort study was conducted. Disease diagnosis followed the regulations of the *International Classification of Diseases, Ninth Revision, Clinical Modification* (ICD-9-CM). ICD-9-CM code 388.2 was used to define SSHL. Medical records that matched with these codes were obtained from the NHIRD between 2000 and 2009 for further analysis. Patients diagnosed with SSHL between January 1, 2000, and December 31, 2009, for > 1 year who had attended ≥2 outpatient visits or received any inpatient diagnosis were included as the SSHL group. Patients diagnosed before 2000, aged <20 years, or diagnosed with dementia before SSHL were excluded. A total of 3731 newly diagnosed patients with SSHL were included, and through propensity score matching at a 1:3 ratio according to age, sex, index year, comorbidities, and medications (Fig. 1), the comparison group was constructed. For patients without SSHL or dementia, the index date was designated as 365 days after the diagnosis date. At last, 3725 patients with SSHL and 11,175 without SSHL were selected. The study was approved by the Institutional Review Board of Kaohsiung Medical University Hospital (KMUHIRB-EXEMPT (II)-20,160,028). All methods were carried out in accordance with relevant guidelines and regulations. Because the patient identifiers were scrambled to the public for research purposes to protect confidentiality, the requirement for written or verbal consent from patients for data linkage study was waived.

### Covariates

Details of the patients’ sex, age, area of residence, insurance premium (≤NT$15,840, NT$15,841–25,000, and ≥ NT$25,001), comorbidities, and medications were obtained from the database. To evaluate the contribution of SSHL to the risk of dementia based on age, the participants were divided into three age groups (< 40, 40–59, and ≥ 60 years). We analyzed comorbidities that are well-known risk factors for dementia: hypertension, hyperlipidemia, diabetes, depression, ischemic heart disease, and cerebral vascular disease, and medications, including anticoagulant agents, antiplatelet agents, statins, antidiabetic agents, and antihypertension agents. Comorbidities were considered present if they were diagnosed between 2000 and 2009 prior to the diagnosis of dementia. Incidences of dementia and all-cause endpoints were defined as the study endpoints. Those who were alive and had no disease on December 31, 2010, were excluded after this time point. Person-year risk, the risk of dementia between the SSHL and comparison group, was deemed to be the time between the patient’s respective endpoint and the date of the diagnosis of SSHL or January 1, 2000 (for the non-SSHL group).

### Outcome variable

Dementia (ICD-9-CM codes 290.1, 290.4, 294.1, 294.2, and 331.0) constituted the study outcome (Fig. 1). A diagnosis of dementia was based on at least two outpatient visits or at least one inpatient visit with a diagnosis of dementia listed on the claims data. Patients were followed from the index date to the earliest occurrence of dementia, death, disenrollment from the national health insurance, or the end of the study date (December 31, 2010), whichever came first.

### Statistical analysis

Incidence rates for dementia per 1000 person-years were defined as the number of patients with dementia divided by person-years at risk. To identify whether SSHL increased the risk of dementia, we calculated the hazard ratios (HRs) and 95% confidence intervals (CIs; adjusted for other predictor variables) using Cox proportional hazards regression analysis. The proportional hazards assumption would be checked using statistical tests and graphical diagnostics based on the scaled Schoenfeld residuals. A plot that shows a non-random pattern against time is evidence of violation of the assumption. If the proportional hazard assumption is violated for some covariate, it is possible to stratify taking this variable into account and use the proportional hazards model in each stratum for the other covariates. With respect to the subgroup analysis, participants were stratified by age (<65 and ≧65 years), and sex to compare the outcome of interest in the specific subgroup. We used the Kaplan–Meier method to analyze the cumulative incidence of dementia between the SSHL cohort and the comparison cohort and investigated the difference with a log-rank test. SAS (version 9.3 for Windows; SAS Institute, Cary, NC, USA) was used for all statistical operations. All *P*-values were 2-sided, and *P* < .05 was considered significant.

## Results

### Patient characteristics

The two cohorts comprised 3725 newly diagnosed patients with SSHL and 11,175 age- and sex-matched controls; their data were extracted from the NHIRD 2000–2010. Table 1 summarizes the cohort characteristics. Most cases of SSHL occurred in the 40–59 age group (40.5%), followed by the ≥60 age group (35%). Most patients were men. No significant differences were observed in the distribution of comorbidities and medication between the SSHL and matched comparison cohorts (Table 1).

**Table 1:** Basic characteristics of the SSHL and comparison cohorts (*N* = 14,900)

Variables	Comparison cohort (*N* = 11,175)	SSHL cohort (*N* = 3725)	*P* value
	N (%)	N (%)	
Age			0.823
< 40	2731 (24.4)	918 (24.6)	
40–59	4484 (40.1)	1508 (40.5)	
≧60	3960 (35.4)	1299 (34.9)	
	Mean (±SD)		
Age (years)	52.1 (±16.4)	52.0 (±16.4)	0.726
Sex			0.977
Female	5199 (46.5)	1734 (46.6)	
Male	5976 (53.5)	1991 (53.4)	
Insurance range			0.640
< NT 15,840	3928 (35.1)	1339 (35.9)	
NT 15,841-25,000	4193 (37.5)	1371 (36.8)	
> NT 25,001	3054 (27.3)	1015 (27.2)	
Comorbidities			
Diabetes	1613 (14.4)	569 (15.3)	0.209
Hyperlipidemia	2358 (21.1)	801 (21.5)	0.603
hypertension	3700 (33.1)	1230 (33.0)	0.920
Depression	1001 (9.0)	323 (8.7)	0.595
Ischemic heart disease	197 (1.8)	74 (2.0)	0.376
Cerebral vascular disease	360 (3.2)	133 (3.6)	0.302
Charlson’s index score			0.723
≦1	8395 (75.1)	2793 (75.0)	
2	1276 (11.4)	414 (11.1)	
≧3	1504 (13.5)	518 (13.9)	
Medications			
Anticoagulant agents	64 (0.6)	24 (0.6)	0.621
Antiplatete agents	366 (3.3)	137 (3.7)	0.239
Antidiabetic agents	1088 (9.7)	386 (10.4)	0.267
Antihypertension agents	2730 (24.4)	935 (25.1)	0.410
Statin	523 (4.7)	190 (5.1)	0.298

### Association of SSHL with risk of dementia

Of the 14,900 patients whose data were analyzed in this study, 442 developed dementia in the follow-up period: 126 (3.4%) in the SSHL cohort and 316 (2.8%) in the comparison cohort (Table 2). The mean (standard deviation) follow-up intervals were 5.20 (2.83) years for the SSHL cohort and 5.55 (2.79) years for the comparison cohort (Table 3). SSHL was associated with increased hazard of developing dementia (HR = 1.39; 95% CI = 1.13–1.71; *P* = .002) in the Cox regression model adjusted for age, sex, area of residence, insurance premium, all comorbidities, and medications (Table 2).

**Table 2:** Risk of all-cause dementia in the comparison and SSHL cohorts

	No. cases	Per 1000 Person year	Crude HR (95%CI)	*P* value	Adjusted HR (95%CI)	*P* value
Comparison cohort	316	5.09	Ref.		Ref.	
SSHL cohort	126	6.50	1.28 (1.04–1.57)	0.019	1.39 (1.13–1.71)	0.002

Adjusted for age, sex, area of residence, insurance premium, comorbidities, history of medication, and Charlson’s index score

**Table 3:** The average follow-up duration and new onset dementia duration of the two cohorts

	Follow-up duration		New onset dementia duration	
	Mean (SD)	*p* value	Mean (SD)	*p* value
Comparison cohort	5.55 (2.79)	< 0.001	3.68 (2.59)	0.287
SSHL cohort	5.20 (2.83)		3.39 (2.43)	

### Stratification by age and sex

To determine whether SSHL is a sex- and age-dependent risk factor for dementia, patients were stratified into four groups by age (< 65 years vs. ≥65 years) and sex (female vs. male). Women and older adults (≥65 years) with SSHL had a higher risk for dementia than the comparison group in the Cox regression models after adjustment for the potential confounding factors (HR = 1.64; 95% CI = 1.22–2.22; *P* = .001 and HR = 1.34; 95% CI = 1.06–1.68; *P* = .013, respectively); women with SSHL who were < 65 years old had the highest risk (2.14, 95% CI = 1.17–4.11, *P* = .022; Table 4).

**Table 4:** Risk of all-cause dementia stratified by sex and age between comparison and SSHL cohorts

	Comparison cohort		SSHL cohort		SSHL cohort Verse Comparison cohort	
	No. cases	Per 1000 PY	No. cases	Per 1000 PY	Adjusted HR (95% CI)	*p* value
Sex						
Female (*N* = 6933)	143	4.93	62	6.90	1.64 (1.22–2.22)	0.001
Male (*N* = 7967)	173	5.24	64	6.16	1.24 (0.93–1.66)	0.138
Age						
< 65 year old (*N* = 11,046)	46	0.98	24	1.60	1.62 (0.98–2.65)	0.058
≧65 year old (*N* = 3854)	270	18.03	102	23.17	1.34 (1.06–1.68)	0.013
Age & Gender						
**Age < 65 & Female** (*N* = 5291)	23	1.02	15	2.08	2.14 (1.17–4.11)	0.022
Age < 65 & Male (*N* = 5755)	23	0.94	9	1.16	1.20 (0.55–2.59)	0.647
**Age≧65 & Female** (*N* = 1642)	120	18.73	47	26.28	1.55 (1.10–2.17)	0.012
Age≧65 & Male (*N* = 2212)	150	17.40	55	21.03	1.23 (0.91–1.68)	0.184

Adjusted for age, sex, area of residence, insurance premium, comorbidities, history of medication, and Charlson’s index score

**Fig. 2:**
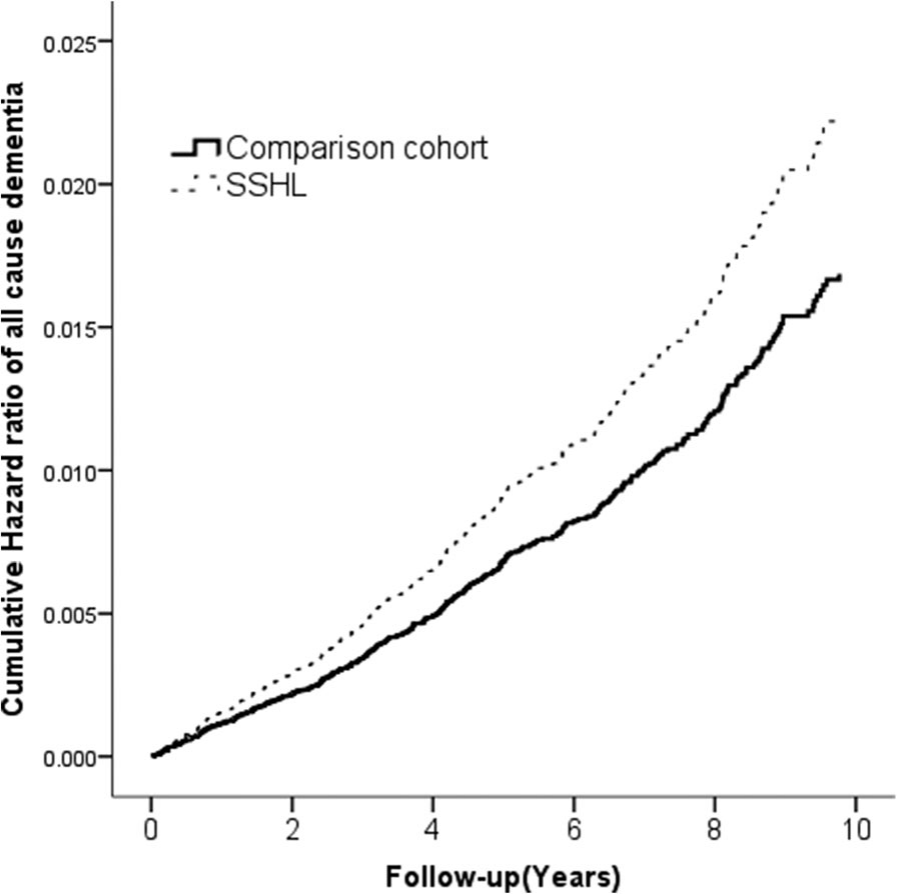
Cumulative hazard ratio of dementia by study groups (SSHL group [dashed line] vs comparison group [solid line]). Modified log-rank test *P* value = .019

The Kaplan–Meier analysis revealed that patients with SSHL had a significantly higher cumulative incidence of dementia than the comparison group (log-rank *P =* .019; Fig. 2).

## Discussion

This population-based cohort study demonstrated SSHL was associated with increased hazard of developing dementia. Even after adjustment for multiple confounding factors, patients with SSHL had a greater risk of developing dementia than those without SSHL. Subgroup analyses revealed that women or older adults (≥65 years) with SSHL had a greater risk of dementia and that the risk was the highest in women with SSHL who were < 65 years old.

No large-scale study has addressed the correlation between SSHL and dementia using propensity score matching. Studies have focused on the relationship between ARHL, such as sensory HL or presbycusis, and the development of Alzheimer disease and the incidence of dementia [6, 7, 18–21]. Patients with HL, based on observation of hearing difficulties during testing or interview, have a faster rate of cognitive decline and are at greater risk of cognitive impairment [22]. This finding corroborates the notion that midlife HL may be responsible for on average 9.1% of dementia diagnoses worldwide [23]; thus, efforts should continue to reduce its impact.

The potential mechanisms underlying the association between ARHL and cognitive decline, particularly increased risk of dementia [19], remain unclear. Although Füllgrabe et al. suggested that audibility and processing effort can bias the cognitive test performance toward cognitive decline [24], Griffiths et al. proposed a mechanism linking auditory cognitive processing in the medial temporal lobe and dementia pathology [25]. Previously, Wayne and Johnsrude summarized the four directional hypotheses for the relationship between ARHL and cognitive decline, namely the cognitive load on perception hypothesis, sensory deprivation hypothesis, information degradation hypothesis, and common cause hypothesis [26]. Of them, the information degradation hypothesis is well supported by cognitive literature [27, 28], and the common cause hypothesis is another plausible mechanism, as it considers multiple sensory modalities (e.g., increased social isolation) and cognition decline concurrently [29]. However, we cannot exclude other physiological processes, such as factors related to the patient’s lineage (e.g., apolipoprotein E [ApoE] status) or a vascular disease causing both HL and dementia. However, we adjusted for vascular disease risk factors, such as hypertension, smoking, and diabetes, in our models, and a preliminary study did not reveal a positive correlation between ApoE status and HL [19]. Alternative variables such as leisure and mental activities were excluded because they do not cause HL and would thus not be relevant confounders in our models. Other specific associations may also exist between dementia and HL, such as different types of HL. SSHL is a unique disorder that often affects only one ear, and 70% of cases are idiopathic. Although many patients with SSHL recover spontaneously (32–65%), the condition’s underlying causes and pathogenesis remain unclear [10]. Moreover, the onset of SSHL is generally in the fifth or sixth decade of life, which is earlier than that of patients with ARHL [12].

SSHL can result from various identifiable causes (e.g., neoplastic, infectious, autoimmune, neurologic, otologic, metabolic, or vascular diseases; ototoxic drugs; and trauma) with many proposed etiologies and risk factors [30]. Postulated causes of idiopathic SSHL include viral cochleitis [31], microvascular events due to a hypercoagulable state [32], and autoimmune disorders [33, 34]. Furthermore, several disease susceptibility genes, such as *ITGB3*, *MTHFR*, *HSP70*, and *PRKCH*, have been studied in association with SSHL. Most of these genes are related to thrombosis, inflammatory response, and free-radical processes or oxidative stress [35]. Accordingly, factors such as metabolism, inflammation, thrombosis, immunology, and oxidative stress may play key pathogenic roles in the onset and development of SSHL [30]. However, these genes may also be related to dementia or other cerebrovascular diseases [14, 36, 37]. Previous studies have consistently reported that patients with SSHL have significantly higher plasma fibrinogen and cholesterol levels than the general population [38, 39]. Elevated plasma fibrinogen and cholesterol levels also contribute to dementia [40, 41]. Moreover, inflammation and immune responses play critical roles in both SSHL and dementia [42, 43]. Elevated serum levels of proinflammatory cytokines, including interleukin 6 (IL-6), tumor necrosis factor-alpha (TNF-α), and C-reactive protein, can cause cognitive impairment [44, 45]. These associations between systemic inflammation and cognitive impairment have been found in all age groups—young [46], middle-aged [47], and older [48] adults. TNF-α and IL-6 concentrations are higher in patients with SSHL than in controls [49, 50]. Taken together, these findings lead us to speculate that SSHL shares a common etiology with dementia through these underlying mechanisms. Additional investigations are required to verify this.

Notably, women with SSHL, especially those < 65 years of age, had a higher risk of dementia than women without SSHL. The epidemiologic data revealed that more women than men had dementia. This agrees with the worldwide data, which indicate dementia is twice as prevalent in women as in men [51]. Although the risk of dementia is related to age, dementia is ultimately deterioration of the brain and not caused by aging alone. Using data from the NHIRD, Lee et al. [52] reported that HL was positively correlated with the risk of dementia, especially in patients aged 45–64 years. However, unlike in our study, they did not stratify the participants by age and sex, which may have confounded the results. Women have higher estrogen levels, which affects their physiology throughout life, including mental health, brain, and cardiovascular health. Estrogen may protect brain cells [53, 54], and higher estrogen levels in aging women may decrease their susceptibility to dementia [55–57]. In our findings, women aged < 65 years with SSHL were most prone to dementia. Assessing the risk of dementia is complicated; understanding the differences in the relationship between dementia and sex and its association with various diseases may help elucidate its causes and the development preventive strategies.

### Strengths and limitations

Our study used a representative nation-based sample and included physicians’ diagnoses of SSHL, a longitudinal analysis, and a wide array of covariates, including general health status and sociodemographic factors. Nonetheless, our study has several limitations. In particular, the NHIRD does not include information on other potential confounders, such as education level, level of cognitive function at baseline, ApoE ε4, stressful life events, body mass index, smoking habits, air pollution exposure, or family history. Furthermore, the follow-up time was short compared with the preclinical phase of Alzheimer disease. Participants who developed dementia were likely already on a trajectory of cognitive decline. Without knowing the participants’ cognitive status, even if we matched SSHL cases to non-SSHL controls, it would be unclear whether SSHL is a consequence of a common underlying pathology that is also related to dementia risk or whether SSHL may aggravate an underlying condition, precipitating dementia. Moreover, although our study revealed that women with SSHL aged < 65 years had the highest risk of dementia, subgroup analysis revealed that there were only 38 dementia cases (23 in the comparison cohort and 15 in the SSHL cohort), indicating the possibility of overfitting. Further studies should explore the association between dementia and sex in the middle-aged population. Finally, this study included only Taiwanese citizens, so our findings may not be generalizable to other countries.

## Conclusion

The present study investigated a possible link between SSHL and dementia development. We observed that patients with SSHL, especially women aged < 65 years, were associated with higher risk of dementia than those without SSHL. Given the rapidly aging population in Taiwan and worldwide, clinicians should be aware of the risk of dementia in patients with SSHL. Further research is required to explore the mechanisms underlying this association.

## Data Availability

The data that support our findings this study are available from the NHIRD, but restrictions apply to the availability of these data, which were used under license for the current study; thus, they are not publicly available. Data are, however, available from the authors upon reasonable request and with the permission of the NHIRD.

## Abbreviations

SSHL: Sudden sensorineural hearing loss
HL: Hearing loss
ARHL: Aged-Related Hearing Loss
NHIRD: National Health Insurance Research Database
NHI: National Health Insurance
(ICD-9-CM: International Classification of Diseases, Ninth Revision, Clinical Modification
HRs: Hazard ratios
CIs: Confidence intervals
ApoE: Apolipoprotein E
